# Post-translational modifications on the metal-sequestering protein calprotectin

**DOI:** 10.1007/s10534-023-00493-x

**Published:** 2023-02-24

**Authors:** Elizabeth M. Nolan, Janet J. Y. Peet

**Affiliations:** Department of Chemistry, Massachusetts Institute of Technology, Cambridge, MA 02139 USA

**Keywords:** Calprotectin, Nutritional immunity, Metal sequestration, Post-translational modification, Methionine oxidation, Disulfide bond formation

## Abstract

Human calprotectin (CP, S100A8/S100A9 oligomer) is an abundant neutrophil protein that contributes to innate immunity by sequestering nutrient metal ions in the extracellular space. This process starves invading microbial pathogens of essential metal nutrients, which can inhibit growth and colonization. Over the past decade, fundamental and clinical studies have revealed that the S100A8 and S100A9 subunits of CP exhibit a variety of post-translational modifications (PTMs). This review summarizes PTMs on the CP subunits that have been detected and highlights two recent studies that evaluated the structural and functional consequences of methionine and cysteine oxidation on CP. Collectively, these investigations indicate that the molecular speciation of extracellular CP is complex and composed of multiple proteoforms. Moreover, PTMs may impact biological function and the lifetime of the protein. It is therefore important that post-translationally modified CP species receive consideration and integration into the current working model for how CP functions in nutritional immunity.

## Introduction

Transition metals are essential nutrients that bacterial pathogens must acquire from the host environment during infection (Palmer and Skaar 2016). To prevent metal acquisition by invading bacterial pathogens, the host innate immune system mounts a metal-withholding response, termed nutritional immunity, and deploys metal-sequestering proteins at sites of infection (Weinberg 1975; Hood and Skaar 2012; Murdoch and Skaar 2022). Calprotectin (CP, S100A8/S100A9 oligomer, MRP8/MRP14 oligomer) is an abundant innate immune protein released by neutrophils that limits bacterial growth by sequestering first-row transition metal ions in the extracellular space (Fagerhol et al. 1980; Dorin et al. 1987; Odink et al. 1987; Andersson et al. 1988; Steinbakk et al. 1990; Sohnle et al. 1991a, b; Clohessy and Golden 1995; Corbin et al. 2008; Zygiel and Nolan 2018, 2019). While a multitude of functional and clinical studies of CP have been pursued since its discovery in 1980 (Fagerhol et al. 1980), major advances in our understanding of its metal-withholding activity have been made relatively recently (Zackular et al. 2015; Zygiel and Nolan 2018, 2019).

**Fig. 1:**
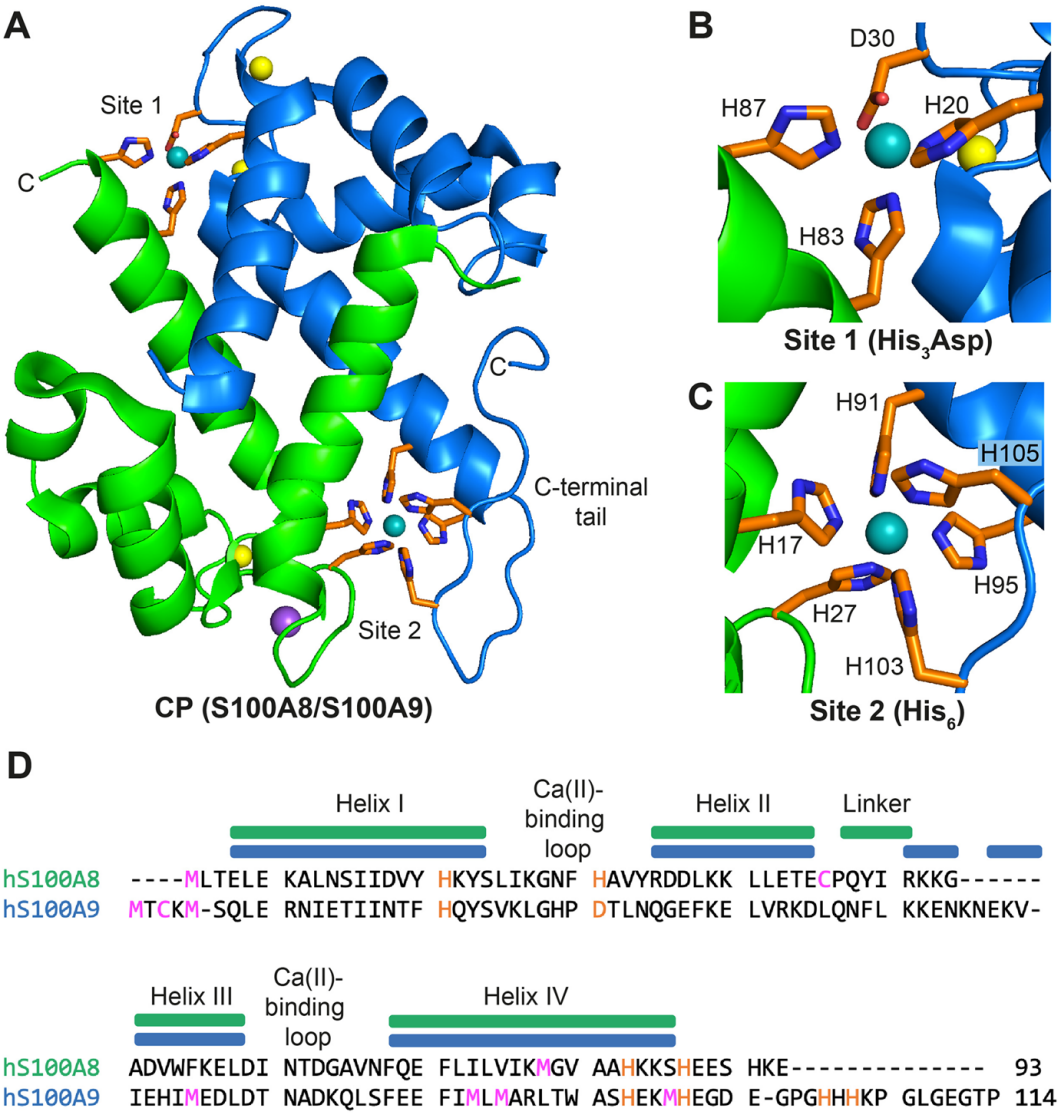
Structural overview of human CP and its metal-binding sites. **A** A S100A8/S100A9 heterodimer unit taken from the crystal structure of the Ni(II)-, Ca(II)- and Na(I)-bound CP-Ser heterotetramer (PDB 5W1F) (Nakashige et al. 2017). **B** Close-up view of the His_3_Asp site. **C** Close-up view of the His_6_ site. S100A8 is green, S100A9 is blue, Ni(II) ions are cyan, Ca(II) ions are yellow, and Na(I) ions are purple. In this structure, the N-terminal EF-hand of S100A8 has a bound Na(I) ion from the buffer. **D** Amino acid sequence alignment of human S100A8 and S100A9. The secondary structural elements are presented above the alignment. The transition-metal binding residues are shown in orange. Met and Cys residues are shown in magenta

Human CP is a heterooligomer of two Ca(II)-binding proteins, S100A8 (α, 10.8 kDa) and S100A9 (β, 13.2 kDa) (Fig. 1) (Hunter and Chazin 1998; Vogl et al. 1999; Strupat et al. 2000). Each S100 subunit contains two EF-hand domains for Ca(II) binding, an N-terminal “noncanonical” EF-hand and a C-terminal “canonical” EF-hand (Gifford et al. 2007). Apo CP is an αβ heterodimer that has two sites for transition-metal binding at the S100A8/S100A9 interface, a His_3_Asp motif (site 1) and a His_6_ motif (site 2) (Körndorfer et al. 2007; Brophy et al. 2012, 2013; Damo et al. 2013). Ca(II) binding to the EF-hands causes two αβ heterodimers to associate and form the (αβ)_2_ heterotetramer (Vogl et al. 1999; Strupat et al. 2000; Adhikari et al. 2020; Silvers et al. 2021). Both the heterodimer and heterotetramer coordinate metal ions at the His_3_Asp and His_6_ sites; however, Ca(II) binding markedly increases the transition metal affinities of both sites and enables multi-metal sequestration (Brophy et al. 2012; Zygiel and Nolan 2018). The His_3_Asp motif sequesters Zn(II) and binds other M(II) with only relatively low affinity; in contrast, the His_6_ site is functionally versatile and sequesters Mn(II), Fe(II), Ni(II), and Zn(II) (Brophy et al. 2013; Damo et al. 2013; Hayden et al. 2013; Nakashige et al. 2015, 2016, 2017). The ability of CP to capture multiple kinetically labile M(II) at the His_6_ site stems from the involvement of the S100A9 C-terminal tail (residues 96–114), which provides two His residues for M(II) coordination, encapsulates the M(II) in the site, and blocks solvent access (Brophy et al. 2013; Damo et al. 2013; Gagnon et al. 2015). Recent studies demonstrate that CP also sequesters Cu from microbial pathogens (Besold et al. 2018; Wang et al. 2019; Zygiel et al. 2019), but further coordination chemistry studies are required to elucidate the Cu-binding properties of CP.

**Fig. 2:**
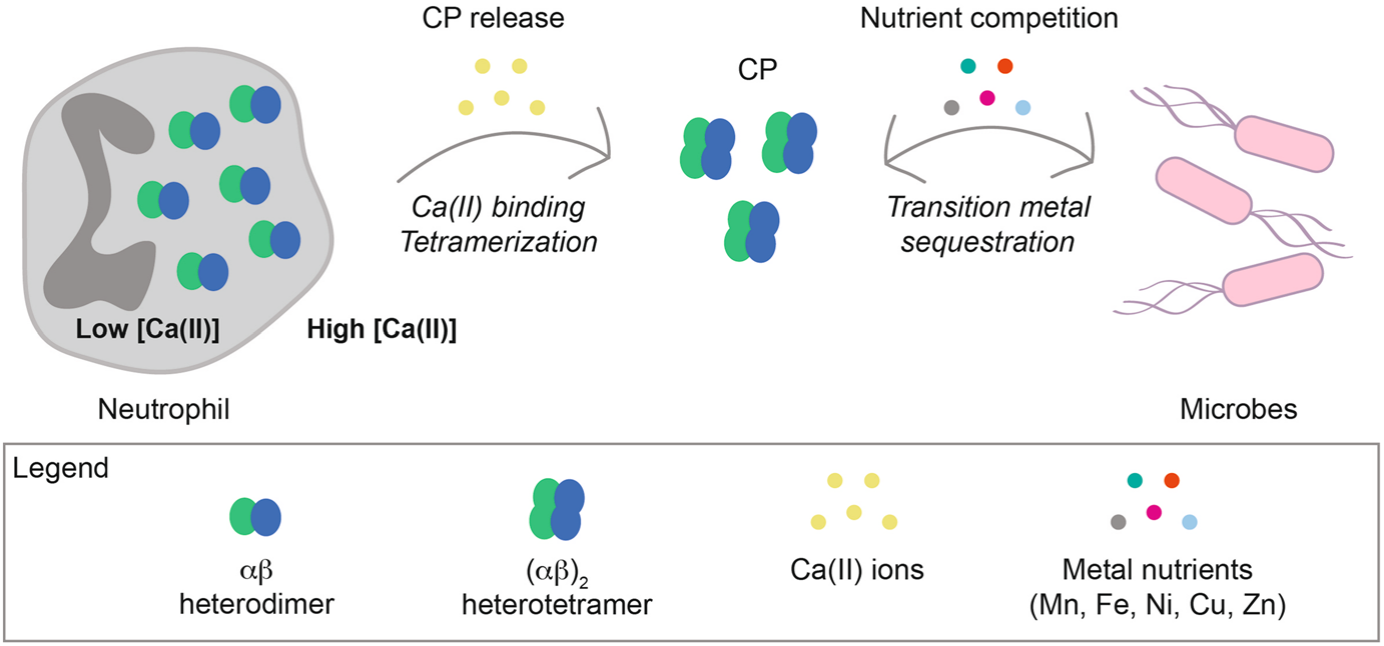
Working model for extracellular metal sequestration by CP. Following release from the neutrophil, CP heterodimers bind Ca(II) in the extracellular space and self-associate to form CP heterotetramers (bound Ca(II) ions not shown), which compete with invading microbial pathogens for essential metal nutrients

The current working model for CP in extracellular metal sequestration begins with neutrophil recruitment to an infection site (Fig. 2) (Zygiel and Nolan 2018). In the cytoplasm under low [Ca(II)] conditions, the CP αβ heterodimer predominates. When CP is released into the extracellular space, which contains high [Ca(II)] (~ 2 mM) (Brini et al. 2013), binding of Ca(II) to the EF-hand domains triggers self-association to form the (αβ)_2_ heterotetramer, which displays enhanced proteolytic stability and metal-binding affinities and is capable of competing with microbial pathogens for multiple M(II) (Brophy et al. 2012; Stephan and Nolan 2016; Zygiel and Nolan 2018). Because CP sequesters multiple M(II), the “relevant” metal-sequestering activity(ies) will depend on metal availability at a given infection site as well as the nutritional requirements of the pathogen(s). This description provides a starting point for continued testing and refinement. For instance, recent studies have shown that the interplay between CP and bacterial secondary metabolites influences metal sequestration and that environmental variables such as local pH impact the metal-sequestering ability of CP (Nakashige and Nolan 2017; Rosen and Nolan 2020; Rosen et al. 2022).

In this review, we focus on advances in defining and understanding the complex molecular speciation of CP in the extracellular space, emphasizing post-translational modifications (PTMs) on the human protein. In recent years, various PTMs on S100A8 and S100A9 have been detected in human specimens (Table 1 and references therein), and there is growing interest in understanding their structural and functional consequences (Lim et al. 2009; Stephan et al. 2018; Hoskin et al. 2019). We highlight two recent studies that investigated oxidative PTMs on CP, including Met oxidation and disulfide-bond formation (Stephan et al. 2018; Hoskin et al. 2019). We intend for this contribution to be a resource for the community and to motivate further consideration and study of post-translationally modified CP in nutritional immunity and beyond.

**Table 1:** Post-translationally modified CP subunits detected in select human and murine specimens^a,b,c^

Subunit	PTM	Sample	Ref.
S100A8	Met sulfoxide (MetO)	Murine kidney tissue Human sputum Human BALF Human saliva Human kidney stones Human nasal mucus Human pimple pus Human plasma	Spraggins et al. (2015)^d^ Gomes et al. (2013) Magon et al. (2015) Cabras et al. (2015) Martelli et al. (2016) Stephan et al. (2018) Stephan et al. (2018) Dubois et al. (2020)
S100A8	Dehydromethionine	Human BALF	Magon et al. (2015)
S100A8	Cys sulfinic acid (–SO_2_H)	Human saliva	Cabras et al. (2015)
S100A8	Cys sulfonic acid (–SO_3_H)	Murine kidney tissue Human sputum Human saliva	Spraggins et al. (2015)^d^ Gomes et al. (2013) Cabras et al. (2015)
S100A8	Cys oxathiazolidine oxide	Human sputum	Gomes et al. (2013)
S100A8	Cys oxathiazolidine dioxide	Human sputum	Gomes et al. (2013)
S100A8	Cys S-nitrosylation	Human saliva	Cabras et al. (2015)
S100A8	Cys S-glutathionylation	Human saliva	Cabras et al. (2015)
S100A8	Disulfide bond (interdimer)	n.f.^e^	
S100A8-S100A9^f^	Disulfide bond (intradimer)	Human saliva Human saliva Human lung infection Human lung cancer Human BALF (CF)^g^	Cabras et al. (2015) Hoskin et al. (2019) Hoskin et al. (2019) Hoskin et al. (2019) Hoskin et al. (2019)
S100A8	Trp oxidation	Human sputum Human saliva	Gomes et al. (2013) Cabras et al. (2015)
S100A8	Proteolysis fragments	Human kidney stones Human BALF	Martelli et al. (2016) Edwards et al. (2022)
S100A9	N-acetylation	Murine spleen Human nasal mucus Human pimple pus	Raftery et al. (1996) Stephan et al. (2018) Stephan et al. (2018)
S100A9(–MTXKM)	N-acetylation	Human serum (arthritis) Human nasal mucus Human pimple pus Human plasma Human plasma	de Seny et al. (2008) Stephan et al. (2018) Stephan et al. (2018) Gao et al. (2018) Dubois et al. (2020)
S100A9	His N-methylation	Murine spleen Murine tissues	Raftery et al. (1996) Davydova et al. (2021)
S100A9	Phosphorylation	Human saliva	Cabras et al. (2015)
S100A9	Met sulfoxide	Human sputum Human saliva Human kidney stones	Gomes et al. (2013) Cabras et al. (2015) Martelli et al. (2016)
S100A9	Cys S-nitrosylation	Human plasma	Dubois et al. (2020)
S100A9	Cys S-glutathionylation	Human saliva	Cabras et al. (2015)
S100A9	Cys S-cysteinylation	Human saliva	Cabras et al. (2015)
S100A9^f^	Disulfide bond (interdimer)	Human saliva Human BALF (CF)^g^	Cabras et al. (2015) Hoskin et al. (2019)
S100A9	Disulfide bond (intrasubunit)	Murine spleen	Raftery et al. (1996)
S100A9	Proteolysis fragments	Human BALF	Edwards et al. (2022)

^a^Includes analyses of fluids, tissues, and kidney stones; does not include cell culture studies, including those using human white blood cells

^b^Cleavage of N-terminal Met by methionine aminopeptidase is not included

^c^Several reports, including de Seny et al. (2008) and Gao et al. (2018), reported oxidized S100A8 or oxidized S100A9 but do not assign the oxidation. These observations are not listed in Table 1

^d^n.f. = not found. The works cited do not report disulfide-linked S100A8–S100A8 species in human or murine samples

^e^This report suggests ~ 12 unique proteoforms of murine S100A8 in kidney tissue following infection with *Staphylococcus aureus*. Murine S100A9 was too large to be detected

^f^Crosslinks of S100A8–S100A9 and S100A9–S100A9 that cannot be reduced by dithiothreitol and are thus not attributed to disulfide linkages have been detected in some samples, including extracts from human arterial extracts, and are not included in Table 1 (McCormick et al. 2005; Gomes et al. 2013; Hoskin et al. 2019). These irreversible crosslinks may be due to HOCl-generated Cys–Lys linkages (McCormick et al. 2005)

^g^Specimens from CF patients

## The CP subunits undergo various PTMs

A number of studies have identified PTMs on the S100A8 and S100A9 subunits detected in specimens from humans including saliva, bronchial lavage fluid (BALF), sputum from cystic fibrosis (CF) patients, serum, plasma, nasal mucus, pimple pus, and kidney stones (Table 1). These PTMs include N-acetylation on S100A9 and truncated (-MTCKM) S100A9, methionine sulfoxidation on S100A8 and S100A9, Cys S-nitrosylation on S100A8 and S100A9, Cys S-glutathionylation on S100A8, Cys oxidation including sulfinic and sulfonic acid formation on S100A8 and disulfide bond formation involving S100A8 and S100A9, Trp oxidation on S100A8, and Thr phosphorylation on S100A9 (de Seny et al. 2008; Gomes et al. 2013; Cabras et al. 2015; Magon et al. 2015; Spraggins et al. 2015; Gao et al. 2018; Stephan et al. 2018; Hoskin et al. 2019; Dubois et al. 2020; Edwards et al. 2022). Evidence for the formation of dehydromethionine and cysteine oxathiazolidine (di)oxide on S100A8 has also been documented (Gomes et al. 2013). In addition to analyses of human fluids and kidney stones, cell culture studies have revealed various PTMs on S100A8 and S100A9. For instance, N-acetylation on S100A9 was found in an early investigation that employed differentiated HL-60 cells (Tobe et al. 1989), and phosphorylation on the C-terminal Thr residue of S100A9 was observed during studies of neutrophils and monocytes (Edgeworth et al. 1989). More recently, S-nitrosylated S100A9 was found following treatment of human peripheral blood monocytes with oxidatively modified low-density lipoprotein and interferon-gamma (Jia et al. 2014), carbonylation on S100A9 was observed in the cytosolic fraction of human phagocytic neutrophil lysates (Wilkie-Grantham et al. 2015), and disulfide cross-linking was detected for CP from cultured neutrophils (Hoskin et al. 2019). Lastly, several PTMs have been detected on murine CP subunits, including Met oxidation on S100A9 in murine kidney tissue and His N^π^-methylation on S100A9 detected in various murine tissues and cell in culture (Spraggins et al. 2015; Daitoku et al. 2021; Raftery et al. 1996; Davydova et al. 2021).

Collectively, these observations demonstrate the existence of a variety of CP proteoforms that arise from individual or combinations of PTMs on the S100A8 and S100A9 subunits. Nevertheless, a few cautionary points are necessary to clarify some limitations of these investigations that can affect interpretation of the data. First, the majority of these studies provide no information regarding the speciation of S100A8 and S100A9 as heterooligomers, homodimers, or possibly other forms (e.g. linked to other proteins (Hoskin et al. 2019)) in the sample at the time of collection. Consequently, whether the PTMs are on a CP heterocomplex is ambiguous. To address this limitation, one recent proteomics study of CP in human plasma sampled from patients suffering septic shock employed immunoprecipitation to isolate the CP heterocomplex prior to analysis by top-down proteomics, which demonstrated a number of PTMs on CP in circulation (Dubois et al. 2020). A second caveat is that some oxidative PTMs—Met oxidation and disulfide bond formation in particular—can occur adventitiously during or after sample collection, leaving uncertainty as to the physiological relevance of the modification. One study recently addressed this concern by spiking samples of nasal mucus and pimple pus with globally ^15^N-labeled CP-Ser [S100A8(C42S)/S100A9(C3S) variant] immediately after collection and monitoring for Met oxidation of ^15^N-S100A8(C42S) and ^15^N-S100A9(C3S) by mass spectrometry (Stephan et al. 2018). Negligible oxidation to the ^15^N-labeled peptides and oxidized CP subunits in the samples were observed, which provided support that Met oxidation occurred prior to sample collection and was physiologically relevant. Lastly, each of these reports of PTMs on S100A8 or S100A9 provides a snapshot of proteoforms in a particular specimen at a particular time. Despite the wealth of information provided by these analyses, dynamic and temporal information about when modified forms arise and disappear is lacking. In what follows, we focus on oxidative PTMs on CP and describe recent biochemical studies that detailed structural and functional consequences of Met oxidation and disulfide bonding on the human protein.

## Oxidative PTMs affect CP structure and function

**Fig. 3:**
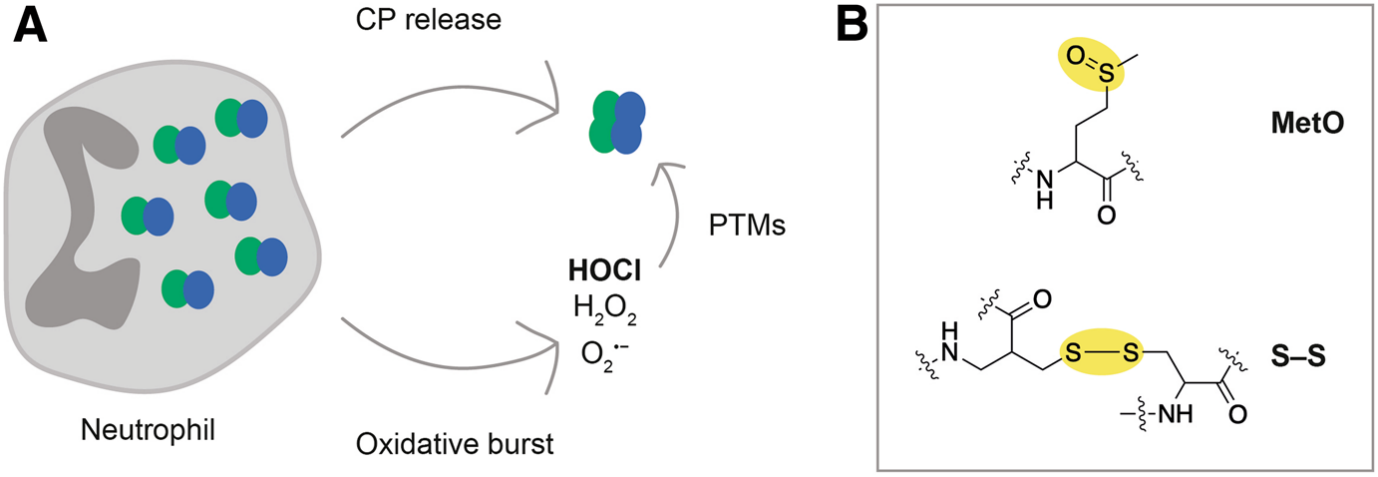
Release of CP and ROS from a neutrophil, and possibility of oxidative PTMs. **A** Cartoon overview. The neutrophil enzymes NADPH oxidase and myeloperoxidase (MPO) sequentially generate O_2_^·–^ and HOCl. These oxidants are released into the extracellular space where HOCl is the likely physiologically relevant oxidant. Generation of H_2_O_2_ occurs via the spontaneous dismutation of O_2_^·–^ or via the sequential reaction of two molecules of O_2_^·–^ with MPO (Winterbourn et al. 2016). **B** Methionine sulfoxide (MetO) and disulfide bonding

Neutrophils produce and release reactive oxygen species (ROS) including superoxide (O_2_^·–^), hydrogen peroxide (H_2_O_2_), and hypochlorous acid (HOCl) during the oxidative burst (Winterbourn et al. 2016). These oxidants can oxidize the side chains of amino acid residues, including Met and Cys. Thus, it was hypothesized that neutrophil-derived ROS post-translationally modify CP. It was also questioned how the resulting oxidative PTMs affect CP structure and function (Fig. 3) (Lim et al. 2009; Stephan et al. 2018; Hoskin et al. 2019). Two recent independent studies focused on the consequences of Met oxidation and disulfide bond formation (Stephan et al. 2018; Hoskin et al. 2019). These investigations showed that such oxidative post-translational modifications on CP result in enhanced susceptibility to host proteases.

## Methionine oxidation case study

**Fig. 4:**
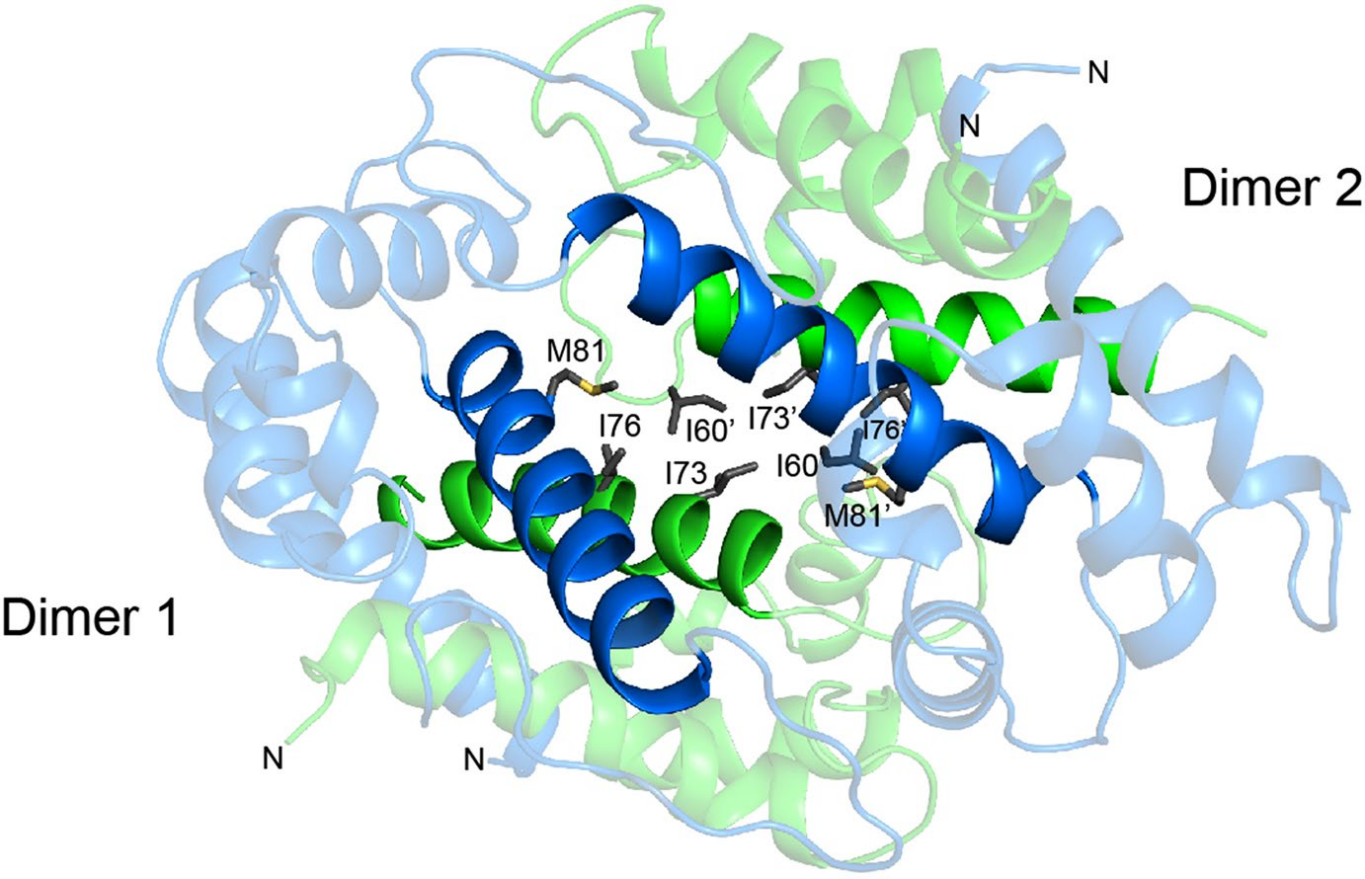
Structure of the Ni(II)-, Ca(II)- and Na(I)-bound CP-Ser heterotetramer (PDB 5W1F) (Nakashige et al. 2017) highlighting the tetramer interface. Select hydrophobic residues that form a hydrophobic network at the tetramer interface are shown in sticks and labeled

Full-length human S100A8 has two Met residues and full-length human S100A9 has six Met residues (Fig. 1d). CP subunits with varying numbers of MetO PTMs have been detected in several human samples (Table 1). To investigate the structural and functional consequences of Met oxidation on CP, samples of the oxidized heterodimer with varying degrees of Met oxidation (hereafter generalized as MetO CP) were prepared chemically using 100–500 mM H_2_O_2_ as an oxidant (Stephan et al. 2018). Although H_2_O_2_ is unlikely to be a physiologically relevant oxidant due to its slow Met oxidation kinetics, it was used in this work because it is a practical reagent for chemically generating MetO CP in the laboratory (Davies 2005; Winterbourn 2013). The CP-Ser variant was used to examine Met oxidation in the absence of cysteine oxidation. MetO CP retained antibacterial activity against laboratory strains of *Escherichia coli* and *S. aureus* and preliminary metal-binding studies showed that it coordinated divalent transition metal ions with high affinity. Nevertheless, biochemical and biophysical examination of MetO CP revealed that Met oxidation had profound consequences for Ca(II)-induced tetramerization, a key feature of CP that confers metal-sequestering ability and protease resistance (Brophy et al. 2012; Stephan and Nolan 2016). In particular, under conditions where 20 equivalents of Ca(II) readily converted the CP αβ heterodimer to the (αβ)_2_ heterotetramer, MetO CP displayed a dynamic equilibrium between heterodimers and heterotetramers. Under these conditions, coordination of a divalent metal ion at the His_6_ site favored tetramerization (*vide infra*), providing a potential explanation for the retained antibacterial activity. Further investigation revealed that oxidation of S100A9(M81) disfavored Ca(II)-induced tetramerization. Notably, S100A9(M81) is located in the tetramer interface along with a number of other hydrophobic residues on S100A8 and S100A9 (Fig. 4). Because MetO is considerably more hydrophilic than Met and calculated to have a hydrophobicity similar to that of lysine (Black and Mould 1991), oxidation of M81 to yield MetO presumably decreases the driving-force for tetramerization.

**Fig. 5:**
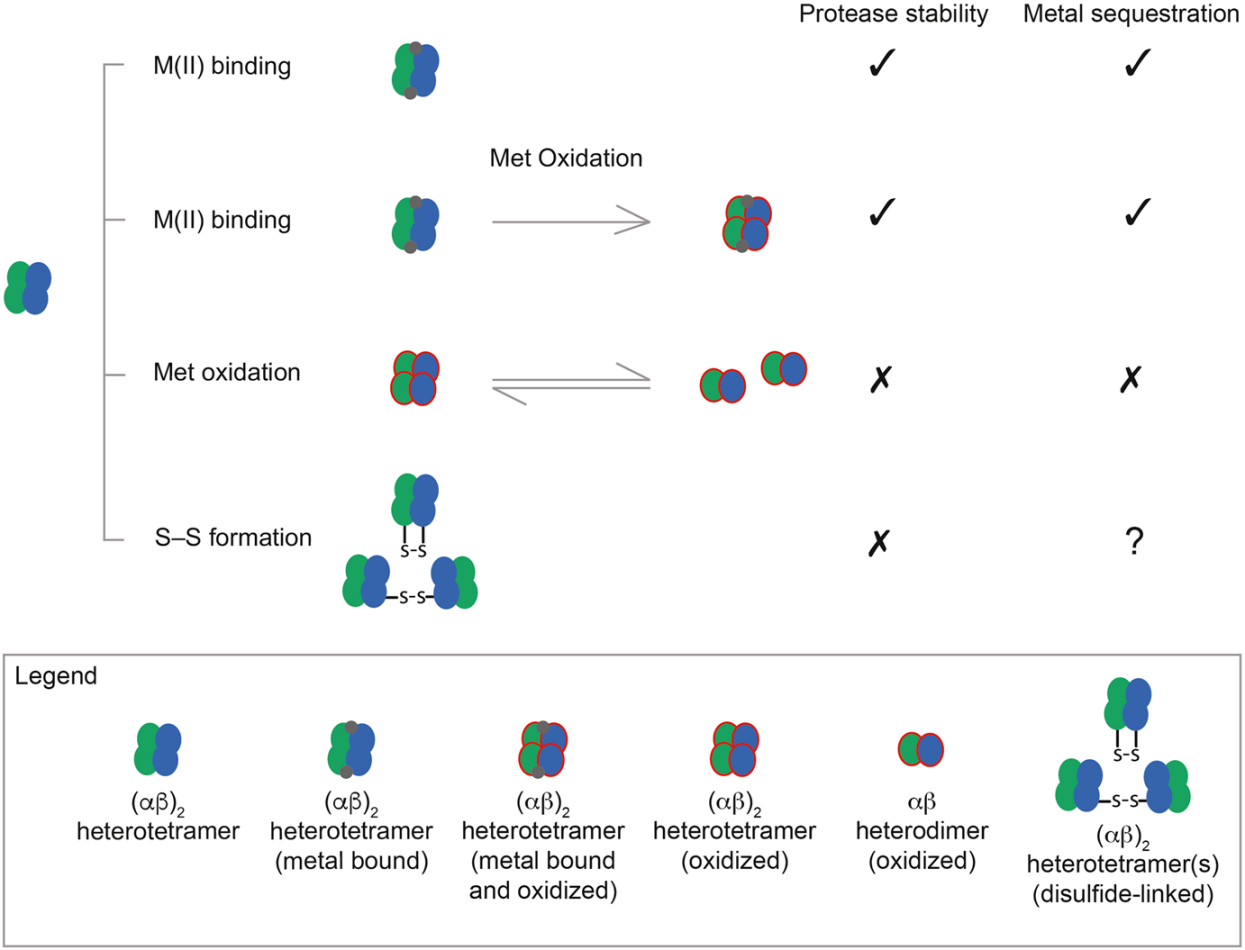
Cartoon overview of oxidative PTM integration into the working model. The possibility of Met oxidation and metal binding occurring simultaneously is not depicted. Further studies of disulfide-linked CP species are required to evaluate the metal-sequestering ability of these proteoforms

A prior study that evaluated tetramer-deficient CP variants revealed that the Ca(II)-bound heterodimer is more susceptible to proteolysis than the Ca(II)-bound heterotetramer (Stephan and Nolan 2016). This prior work, combined with the observation that oxidation of S100A9(M81) disfavored tetramerization, led to the hypothesis that Met oxidation increases the proteolytic susceptibility of CP (Stephan et al. 2018). Indeed, protease degradation assays performed in the presence of excess Ca(II) revealed that MetO CP was more rapidly degraded than CP by extracellular host serine proteases such as trypsin, chymotrypsin, and human neutrophil elastase. In addition to Ca(II)-induced tetramerization, coordination of a divalent metal ion in the His_6_ site causes CP heterodimers to self-associate and form heterotetramers (Stephan and Nolan 2016). The study of tetramer-deficient CP variants also demonstrated that a M(II) such as Mn(II) or Fe(II) bound at the His_6_ site could promote tetramerization and, consequently, recover protease stability (Stephan and Nolan 2016). Likewise, Mn(II) or Fe(II) bound at the His_6_ site of MetO CP recovered tetramerization and protease stability. Based on these findings, this PTM was integrated into the working model for CP in nutritional immunity, where Met oxidation modulates the lifetime of extracellular CP (Fig. 5). Because transition-metal-bound MetO CP retains the ability to tetramerize and resist proteolysis, these metal-bound forms retain their metal-withholding function, which is presumably beneficial to the host. Because CP also mediates pro-inflammatory signaling (Stríz and Trebichavský 2004; Vogl et al. 2007), it was also proposed that degradation of MetO CP without bound transition metal ions serves as a way to dampen the signal. Further investigation of this notion is warranted.

## Disulfide-linked CP case studies

**Fig. 6:**
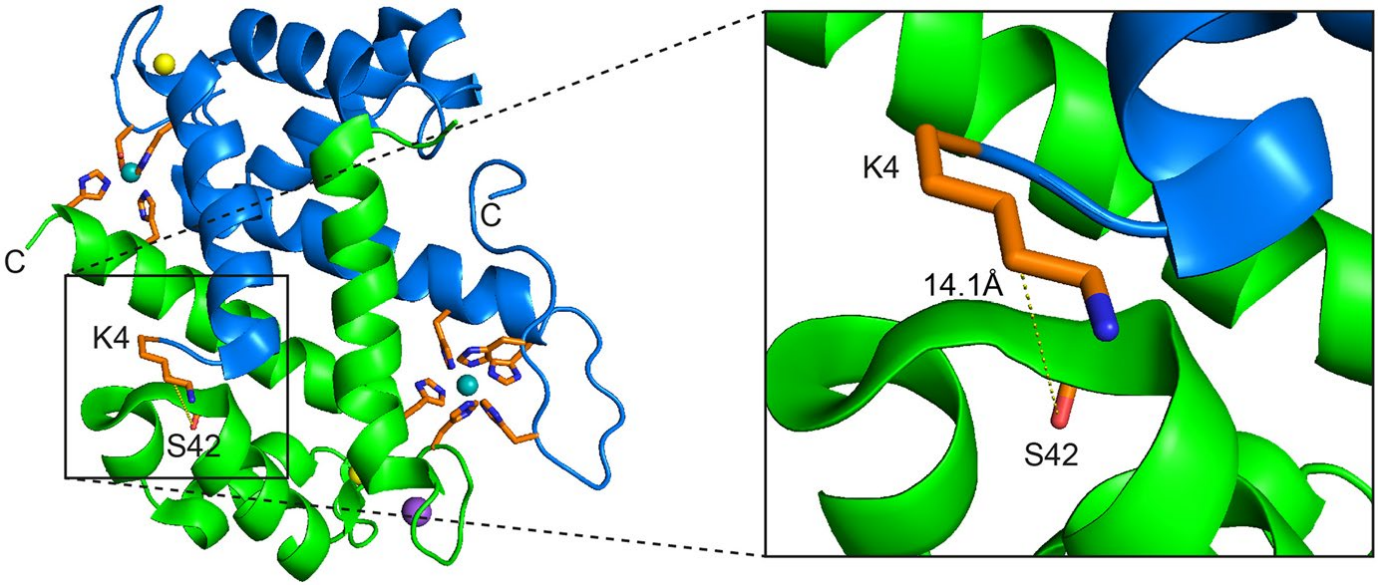
C42 of S100A8 and C3 of S100A9 can be in close proximity as illustrated by the crystal structure of Ni(II)-, Ca(II)- and Na(I) bound CP-Ser (PDB 5W1F) (Nakashige et al. 2017). One heterodimer unit is shown. The N-terminus of S100A9 is disordered in this structure. Consequently, the K4 sidechain was built in using PyMOL. In this depiction, the distance from the γ-carbon atom of K4 to the γ-oxygen atom of S42 is 14.4 Å. A similar distance of 13.3 Å was found in the crystal structure of Mn(II)-, Ca(II)-, and Na(I)-bound CP-Ser (PDB 4XJK) (Gagnon et al. 2015)

Each human CP subunit has one Cys residue, C42 of S100A8 and C3 of S100A9 (Fig. 1d). Two recent studies examined the formation and proteolytic stability of disulfide-linked CP species (Stephan et al. 2018; Hoskin et al. 2019). In a preliminary biochemical investigation of disulfide-bonding in CP, the protein was exposed to 100 μM H_2_O_2_, which afforded disulfide-bond formation without methionine oxidation (Stephan et al. 2018). Western blot analysis using a non-reducing gel revealed the presence of species with S100A9–S100A9, S100A9–S100A8, and S100A8–S100A8 linkages, with the S100A8–S100A8 linked species being a minor species. The S100A9–S100A9 and S100A8–S100A8 species can only arise from S100A9(C3)–S100A9(C3)ʹ and S100A8(C42)–S100A8(C42)ʹ linkages between heterodimers, respectively. The S100A9–S100A8 species could result from S100A9(C3)–S100A8(C42) disulfide bonding between heterodimers or within a heterodimer, a detail that the Western blot analysis could not address. Western blot analysis of time-course studies revealed the conversion of the mixture of species to the S100A8–S100A9 species over time, indicating that disulfide linkages involving C42 of S100A8 are most stable. Indeed, further analysis by analytical size exclusion chromatography and mass spectrometry of disulfide-linked CP mixtures arising from a 23-h incubation of CP with 100 μM H_2_O_2_ and excess Ca(II) ions revealed that the predominant species was a Ca(II)-bound heterotetramer with at least one S100A8–S100A9 “intradimer” disulfide bond. This result was striking because the possibility of a disulfide linkage within a CP heterodimer was largely overlooked in all prior work; an early study of the CP heterodimer concluded such a linkage was not possible based on a homology model (Hunter and Chazin 1998). Nevertheless, examination of available CP-Ser crystal structures that were subsequently reported showed that the N-terminus of S100A9 can be in close proximity to S100A8(C42) (Fig. 6) (Körndorfer et al. 2007; Nakashige et al. 2017), allowing C42 of S100A8 and C3 of S100A9 to form the intradimer disulfide linkage. Studies performed in the absence and presence of Ca(II) revealed that (i) Ca(II) ions altered the disulfide reactivity, which was attributed to a Ca(II)-induced structural change that impacts the accessibility of C42 of S100A8 because this residue is located in the linker region between the N- and C-terminal EF-hands; and (ii) the disulfide-linked CP species underwent Ca(II)-induced oligomerization. This study, which was done in conjunction with the examination of MetO CP described above, also examined the proteolytic stability of disulfide-linked CP species and found that these proteoforms were more rapidly degraded than CP without disulfide bonds. Similar to Met oxidation, disulfide bonding was proposed to modulate the lifetime of CP (Fig. 5). Evaluation of the isolated disulfide-linked CP species, including interrogating coordination chemistry and function, is needed to fully integrate these proteoforms into the working model for CP in nutritional immunity.

Subsequently, a report detailed the formation of disulfide-linked CP species in neutrophil culture (Hoskin et al. 2019). Western blot analyses of intracellular and extracellular fractions from stimulated neutrophils revealed disulfide-linked CP species predominantly in the extracellular material; minimal disulfide-linked CP species were detected in the intracellular material. Moreover, when the oxidant scavenger methionine was added to the culture medium, the amount of disulfide-linked CP species was markedly reduced. Strikingly, the predominant disulfide-linked CP species in the extracellular fraction contained the S100A8–S100A9 linkage. Collectively, these observations support the working hypothesis that neutrophil-generated ROS oxidize CP in the extracellular space (Fig. 3). Further investigation revealed that both NADPH oxidase and myeloperoxidase were necessary for formation of the S100A8–S10A9 linked species, providing strong support for neutrophil-generated HOCl being the primary oxidant. Examination of purified CP treated with HOCl revealed that both S100A9–S100A9 and S100A8–S100A9 linked species were formed in the presence of stoichiometric HOCl, and that increasing the HOCl concentration increased the proportion of S100A8–S100A9 species to the S100A9–S100A9 species in the sample. LC–MS analysis also revealed that HOCl oxidized Met residues in these samples. Further evaluation of the HOCl-generated oxidized CP species showed that oxidation disrupted the quaternary structure and resulted in enhanced susceptibility to proteolysis. The consequences of exposing CP to hypothiocyanous acid (HOSCN), which reacts selectively with Cys residues, was also examined in this work and a subsequent investigation (Hoskin et al. 2019; Edwards et al. 2022). Treatment of CP with HOSCN resulted in the formation of S100A8–S100A9 linked species—no S100A9–S100A9 linked species were detected—and increased susceptibility to proteolysis (Hoskin et al. 2019; Edwards et al. 2022). The agreement of these two independent studies on disulfide linkages in CP and the consequences of this PTM on proteolytic stability along with the detection of disulfide-linked CP species in various human samples provides a strong foundation for further studies of these particular proteoforms.

## Perspectives

The two recent reports of oxidative modifications on the CP subunits provide a compelling picture that MetO and disulfide bond formation have marked consequences on CP structure and stability. Further elucidating the ramifications of these PTMs and others on the biophysical properties, biological function, and fate of CP is important. More broadly, with numerous PTMs on CP detected since the mid-1990s, it becomes apparent that the complex molecular speciation of CP must be considered not only from the standpoints of various oligomers and metal-bound forms, but also from the standpoint of covalent modifications. All of these structural features, in isolation and in combination, warrant consideration and integration into the working model for CP in nutritional immunity.

Beyond understanding how various PTMs affect CP structure and function, many additional questions exist. Specific to the examples described above, MetO and disulfide bond formation are reversible PTMs, but it is unknown whether methionine sulfoxide reductases and thioredoxins reverse these modifications. In addition to the oxidative PTMs highlighted in this review, other PTMs detected in murine and human samples are fascinating and require investigation. For instance, it was recently discovered that the N^π^-methyltransferase METTL9 methylates His107 of murine S100A9 (Daitoku et al. 2021; Davydova et al. 2021,). This observation is striking because His107 is one of the six His residues that compose the His_6_ site of murine CP and the N^π^-methylhistidine modification would disrupt the M(II) coordination sphere (Hadley et al. 2019). Similar to MetO and disulfide bond formation, histidine methylation is a reversible PTM and it is intriguing to consider the possibility that a demethylase may allow murine CP to regain its metal-sequestering capacity. Lastly, although this review considers CP from the standpoint of its roles in the host-microbe interaction and nutritional immunity, CP has multiple functions beyond metal-withholding, including contributing to the progression of inflammation and cancer (Stríz and Trebichavský 2004; Goyette and Geczy 2011; Jo et al. 2021; Mondet et al. 2021). The consequences of PTMs on CP in these pathologies also warrant future exploration.
